# Evaluation of the Reduction of Skin Hyperpigmentation Changes under the Influence of a Preparation Containing Kojic Acid Using Hyperspectral Imaging—Preliminary Study

**DOI:** 10.3390/jcm12072710

**Published:** 2023-04-04

**Authors:** Iga Wawrzyk-Bochenek, Mansur Rahnama, Martyna Stachura, Sławomir Wilczyński, Anna Wawrzyk

**Affiliations:** 1 Department of Basic Biomedical Science, Faculty of Pharmaceutical Sciences in Sosnowiec, Medical University of Silesia, Kasztanowa 3, 41-205 Sosnowiec, Poland; 2 Chair and Department of Oral Surgery, Medical University of Lublin, Chodźki 6, 20-093 Lublin, Poland; 3 Silesian Park of Medical Technology Kardio-Med Silesia in Zabrze, M. Curie Skłodowskiej 10C, 41-800 Zabrze, Poland

**Keywords:** skin hyperpigmentation, kojic acid, hyperspectral camera, quantitative analysis

## Abstract

**Aim**: The aim of this study was to demonstrate the effects of using a preparation containing kojic acid on skin hyperpigmentation using hyperspectral imaging, which enables a quantitative assessment of the effect of the preparation used on the reduction of skin discoloration. **Materials and methods**: Preliminary studies were carried out on 12 patients with post-acne skin. A hyperspectral camera with a spectral range of 400–1000 nm was used to image skin hyperpigmentation before and after the application of 3% kojic acid. Hyperspectral profiles were analyzed, and image analysis and processing methods were applied. **Results**: Studies performed using a hyperspectral camera have shown that kojic acid reduces skin discoloration by increasing skin brightness in 75% of patients tested, reducing skin contrast in approximately 83% and increasing skin homogeneity in approximately 67% of patients.

## 1. Introduction

Hyperpigmentation of the skin associated with disturbed melanin synthesis and/or its incorrect distribution in the skin is a common dermatological and cosmetic problem affecting the appearance of a patient’s skin. Most often, they take the form of spots with a clearly darker color than healthy skin and are located in areas exposed to constant UV radiation [1].

Depending on the location of the accumulated pigment in the skin, discoloration can be divided into: epidermal (superficial), resulting from the accumulation of excessive amounts of melanocytes in the epidermis or overproduction of melanin; dermal (deep), arising when an excessive amount of melanin accumulates in the papillary layer of the dermis; and mixed, arising when the pigment accumulates in both the epidermis and the dermis [2,3,4].

Skin color is genetically determined and depends on the thickness of the epidermis, the content of hemoglobin and melanin. The main thing is the balance between the content of eumelanin (black–brown pigment) and pheomelanin (yellow–red pigment) [5].

Eumelanin has a photoprotective effect, limiting the penetration of UV radiation into the epidermis and capturing reactive oxygen radicals. Eumelanin is much brighter in terms of protection against UV radiation and its effects than pheomelanin [6,7,8].

The type of color depends on the quantitative ratio of eumelanin and pheomelanin, as well as the number, activity of melanocytes and the content of melanosomes. In the case of chronic inflammation, skin eruption and physiological processes occurring within the skin with age, the distribution of the pigment is uneven. Most discoloration is not a health pathology, and after the exclusion of melanoma, it can be subjected to various cosmetology treatments (chemical peels; microdermabrasion; cryotherapy; the introduction of brightening substances by means of physical agents such as sonophoresis, iontophoresis and electroporation; the introduction of brightening substances by means of punctures such as mesotherapy; and laser therapy such as with a Q-Switch ruby laser, Q-Switch Nd-YAG laser, pulsed dye laser (PDL), Q-Switch alexandrite laser and IPL) [9].

Post-inflammatory hyperpigmentation may persist for several weeks or even years. Inflammation stimulates the production of prostaglandins, leukotrienes and thromboxanes, which increase the activity of melanocytes in the epidermis and thus stimulate increased melanin synthesis. These types of discoloration occur as irregular, dark patches of skin where previous injury or inflammation has occurred [10].

In order to reduce skin discoloration, chemical products are usually used that reduce the activity of tyrosinase, an enzyme necessary for melanin biosynthesis. One of the substances used for this purpose is kojic acid [11,12].

Kojic acid (5-hydroxy-2-hydroxymethyl-4H-pyran-4-one, KA) belongs to the group of organic acids. It is obtained during the process of aerobic fermentation from fungi such as: Aspergillus flavus, Aspergillus oryzae, Aspergillus parasiticus and Aspergillus tamarii or is made from soy sauce or rice wine. The name of the acid comes from the fungus Aspergillus oryzae, which in Japan is called “Koji” [12,13]. Kojic acid reduces discoloration by inhibiting tyrosinase activity. Kojic acid captures copper ions, preventing the activation of tyrosinase, thus preventing the formation of melanin [12,14]. Due to its tyrosinase-inhibiting activity, KA is considered one of the most effective skin lightening agents in the beauty industry [15]. Kojic acid is a hydrophilic molecule containing a reactive gamma-pyrone ring with weak acidity. It is in the form of a white, crystalline powder [12,14]. It also has antioxidant properties [16,17] and is used as a substitute for hydroquinone (HQ) for skin lightening by the cosmetics industry [18]. KA also has anti-inflammatory properties [19].

In the assessment of skin lesions such as hyperpigmentation and in assessing the effectiveness of their reduction, hyperspectral imaging can be used more and more often, which allows for a reliable, objective and accurate measurement of changes after various therapies [20]. Hyperspectral imaging is a hybrid technique of traditional imaging and spectroscopy. A hyperspectral image consists of hundreds of images (depending on spectral resolution) that are recorded at specific wavelengths. High spectral resolution provides the possibility of quantitative and qualitative interpretation. Using a hyperspectral camera, data can be recorded covering the range of electromagnetic radiation from infrared, through the visible range, to ultraviolet [21,22]. Hyperspectral imaging is characterized by non-invasive measurement and allows you to record data for the entire object, regardless of its size. The obtained digital image is the basis for further analyses and searches for spectrally different objects [22]. In hyperspectral imaging, light is captured by a lens and separated into different spectral lengths using a prism or a diffraction grating. The basis of the hyperspectral camera is a detector array that reads information adequate to the length of the recorded radiation [23]. There are sensors on the matrix of optical filters, thanks to which pixels can selectively record data for single wavelengths. This enables the recording of images in a wide spectral range [20]. With such imaging, the spatial information of the image can be combined with the chemical information of the spectrum. The images taken with a hyperspectral camera are three-way datasets, one representing the spectral variables and two depicting the spatial attributes of the image [23,24].

Hyperspectral imaging is increasingly used by researchers in cosmetology and dermatology, e.g., to monitor skin reconstruction after injuries or melanin production [20]. The advantages of hyperspectral imaging are: non-contact, the ability to analyze the entire object simultaneously and the ability to assess the minimum and maximum reflectivity. The disadvantages of this method include: longer measurement time and a more complex process of analyzing the results [25].

The aim of this study was to demonstrate the in vivo effects of the use of a preparation containing kojic acid on skin hyperpigmentation using hyperspectral imaging, which enables a quantitative assessment of the effect of the preparation used on the reduction of discolorations.

## 2. Materials and Methods

### 2.1. Patients

The preliminary study included a group of 12 volunteers, which consisted of women aged 25–35. The criteria for exclusion from the study were: the use of retinoids up to 12 months before the study, active inflammation of the skin and pregnant women. All patients had II or III fitzpatrick phototype. Each of the volunteers was marked with numerical codes from 1 to 12. Written consent for the procedure was obtained from the patients. All patients gave written informed consent. The study protocol was in line with the Declaration of Helsinki.

As the ROI—Region of Interest—treatment area, a 3 × 3 cm fragment of the cheek that was covered by hyperpigmentation was designated for each patient (the changes were homogeneous, post-inflammatory and post-acne). The changes were verified by a dermatologist.

The limitation of this method is access to patients classified as having relatively homogeneous skin pigmentation characteristics. People with different skin phototypes were not taken into account in order to maintain the homogeneity of the samples. Men were not included in the study because the characteristics of men’s skin differ from women’s.

### 2.2. Methods

#### 2.2.1. The Procedure of the Applied Treatment

Before the application of kojic acid, make-up removal and toning were applied. A pre-peeling was then applied to evenly penetrate the kojic acid. Lip red, nostrils secured with Vaseline. Kojic acid from Bingospa, Poland with a pH of 1.8 in powdered form was used. A solution of 3% kojic acid in saline was prepared (in a glass beaker). The solution prepared in this way was applied to the skin in a thin layer using a brush. Apis neutralizer, Poland, was used to neutralize the applied acid. The neutralizer was applied in an even layer with a brush. Left for 2 min. The neutralizer was then emulsified (about 1 min) and then removed with a spatula and cotton buds. The application according to the procedure was repeated 4 to 6 times at 2-week intervals. ROIs were exposed to acid for 1 to 5 min depending on skin reaction. In the first procedure, acid was applied for 1 to 2 min. Two consecutive applications of 3 min, 3 consecutive applications of 5 min.

#### 2.2.2. ROI Images in Visible Light

ROI images were documented in RGB using the Fotomedicus clinical skin photography system, Elfo, Poland. This kit enables reproducible acquisition of skin images in cross-polarized light.

#### 2.2.3. Hyperspectral Imaging

In order to quantitatively identify the content and distribution of skin chromophores (mainly melanin), the SPECIM IQ hyperspectral camera, SPECIM, Finland, was used, which allowed the acquisition of skin images in the spectral range of 400–1000 nm with a spectral resolution of 2.9 nm and a spatial resolution of 512 × 512 at the physical size of pixel size of 17.58 × 17.58 μm. Images were taken using a tripod at a distance of approximately 75 cm using a 21 mm focal length lens.

During the study with the use of kojic acid, hyperspectral data before and after a series of treatments were recorded for each patient.

The images were recorded together with the reflectance pattern using an optimized lighting system containing two incandescent lamps with flat spectral characteristics in the wavelength range of the camera, i.e., 400–1000 nm. The integration time for the applied lighting system was from 12 to 19 ms for a single frame, which for 204 frames gave the total registration time of the hyperspectral cube below 4 s. The time of image acquisition (indirectly, the intensity of light) was very important, because image registration longer than 5 s was associated with the formation of artifacts related to the volunteers’ micromovements.

#### 2.2.4. GLCM

A quantitative analysis of the obtained images was also carried out using image analysis and processing algorithms, including GLCM analysis. GLCM analysis, i.e., gray level co-occurrence matrix, determines how many times an image pixel with a given brightness is adjacent to a pixel with a different brightness. GLCM algorithms allow for numerical characterization of image textures by calculating the frequency of coexistence of pixel neighborhoods with operator-specified gray level differences [25].

The GLCM analysis consists of contrast and homogeneity. These parameters are negatively correlated with each other. In the GLCM matrix, the number of columns and rows is equal to the number of gray levels (G). Image analysis can be conducted in different directions: vertically (90°), horizontally (0°) and diagonally (45 or 135°). Before generating the matrix, you must specify the offset d, i.e., the distance between pixels, and the angle θ at which they will be analyzed. In this work, pixels in the horizontal direction (θ = 0°) located in the immediate vicinity (d = 1) were studied. In practice, this means that the *x* row of the matrix and the *y* column contain information about how often a pixel with a certain level of gray (brightness) is located directly to the right of a pixel with a different, defined level of gray. This numerically identifies the element P of the matrix, which informs the frequency of two pixels separated by a distance (Δ*x*, Δ*y*) in a given neighborhood.

GLCM analysis has an advantage over other methods of image analysis and processing in that it not only identifies the number of pixels of a certain quality, but also the relationships between them in n-dimensional space, where n is the number of gray levels.

In summary, GLCM contrast identification can quantify whether an image is homogeneous or not. For the proposed research model, the contrast between the tested pixel and its neighbor on the right was identified, according to the formula:

$$\underset{i,j}{\sum }{\left|i-j\right|}^{2}\;p\left(i,j\right)$$

where:

*i* is the brightness of the tested pixel;
*j* is the neighboring pixel brightness.

A derivative of GLCM contrast is image homogeneity. In the proposed research model, homogeneity is understood as:

$$\underset{i,j}{\sum }\frac{p\left(i,j\right)}{1+\left|i-j\right|}$$

where:

*i* is the brightness of the tested pixel;
*j* is the neighboring pixel brightness.

Homogeneity, similarly to GLCM contrast, is used to evaluate image homogeneity. The higher the homogeneity, the more homogeneous the image is—it contains fewer adjacent pixels with different levels of gray, which corresponds to areas of different density.

The idea of using a hyperspectral camera to assess the content and distribution of melanin before and after a series of treatments using kojic acid was as follows:

The maximum, average and minimum ROI reflectances for each volunteer were assessed. The greater the difference between the maximum and minimum value, the lower the homogeneity of hemoglobin distribution before and after a series of treatments.

The hyperspectral profile (average reflectance) was compared for individual volunteers before and after a series of IPL treatments in the ROI area. On this basis, it was possible—by determining the reflectance coefficient for a given wavelength in the ROI area—to determine the melanin content before and after a series of procedures.

#### 2.2.5. Statistical Analysis

On the basis of the results obtained from hyperspectral imaging, graphs with quantitative, brightness, contrast and homogeneity analysis were prepared. The obtained data were subjected to statistical analysis using Microsoft Excel 2010 and a database necessary to carry out the statistical analysis was developed. In order to determine the differences in the level of significance in the results before and after the application of kojic acid, the Wilcoxon order of pairs test was used.

## 3. Results

### 3.1. Imaging

**Figure 1:**
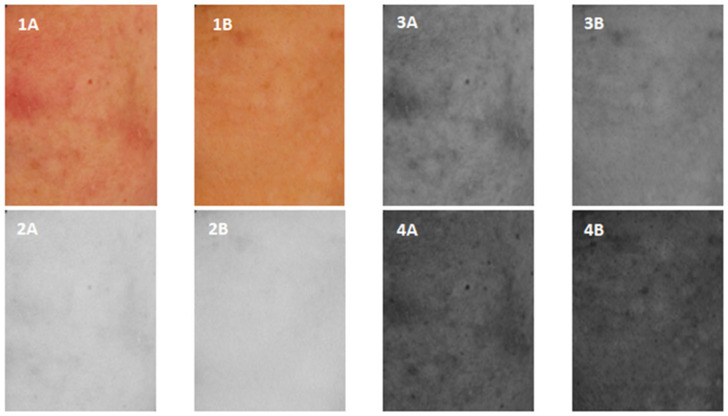
Volunteer 1st ROI images captured in cross-polarized light before and after a series of treatments (**1A**,**2A**,**3A**,**4A**) and after a series of kojic acid treatments (**1B**,**2B**,**3B**,**4B**) for RGB channels ((**1A**) and (**2B**), respectively), red (R) (**2A**,**2B**), green G (**3A**,**3B**) and blue B (**4A**,**4B**).

Figure 1 shows example photos of volunteer No. 1 recorded in cross-polarized light, before a series of treatments (Figure 1(1A,2A,3A,4A)) and after a series of treatments using kojic acid (Figure 1(1B,2B,3B,4B)) for the RGB channels (Figure 1(1A), respectively) and Figure 1(2B)), red (R) (Figure 1(2A,2B)), green G (Figure 1(3A,3B)) and blue B (Figure 1(4A,4B)). Even a cursory visual analysis of the photos shows a significant reduction in erythema.

### 3.2. Quantitative Analysis

Quantitative analysis and plots were also performed for the brightness, contrast and homogeneity of the ROI treated with kojic acid. The mean values of the results, along with the standard deviation, are presented in Table 1.

The median value of facial skin brightness after applying kojic acid increased statistically significantly by 15.50, including the upper quartile by 3.5 and the lower one by 3.0.

The analysis of changes in each person subjected to the procedure showed that the brightness of the skin increased in 75% of patients, and in 25% there was an opposite effect and there was a decrease in the brightness of the facial skin.

**Figure 2:**
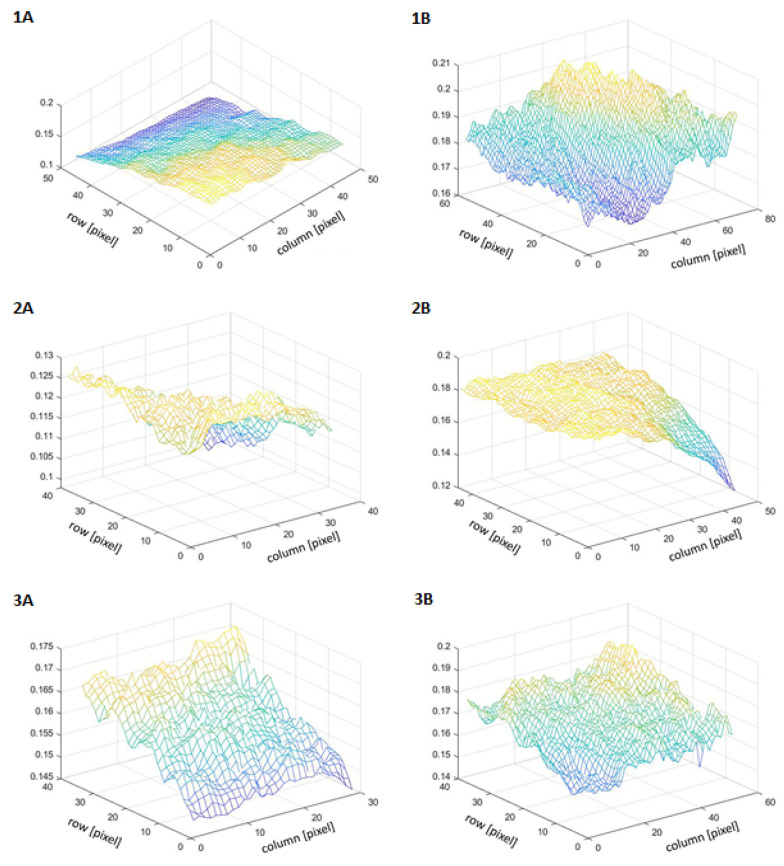
Brightness of the ROI image before the treatment with kojic acid (patient 1—(**1A**), patient 2—(**2A**), patient 3—(**3A**)) and after the treatment with kojic acid (patient 1—(**1B**), patient 2—(**2B**), patient 3—(**3B**)) in 3D space.

The brightness of the examined areas was also visualized in 3D space. The *y*-axis is labeled with the brightness value and the *x*- and *z*-axis are labeled with pixels (Figure 2).

The value of the median GLCM contrast (the so-called gray level co-occurrence matrix) of the facial skin increased by 0.89, and the upper and lower quartiles by 0.31. The described changes in skin contrast after treatment with kojic acid are statistically significant (*p* < 0.05).

The analysis of changes in GLCM contrast in each person subjected to the procedure showed that skin contrast decreased in approximately 83% of the probands, and in approximately 13% the opposite effect occurred and the contrast of the facial skin increased. The above-described changes in skin contrast after treatment with kojic acid are statistically significant (*p* < 0.1).

The median GLCM facial skin homogeneity value increased by 0.06, the upper quartile by 0.017 and the lower quartile by 0.029. A detailed analysis of changes in GLCM homogeneity in each treated patient showed that skin homogeneity increased in approximately 67% of subjects, and the opposite effect occurred in approximately 33%.

**Figure 3:**
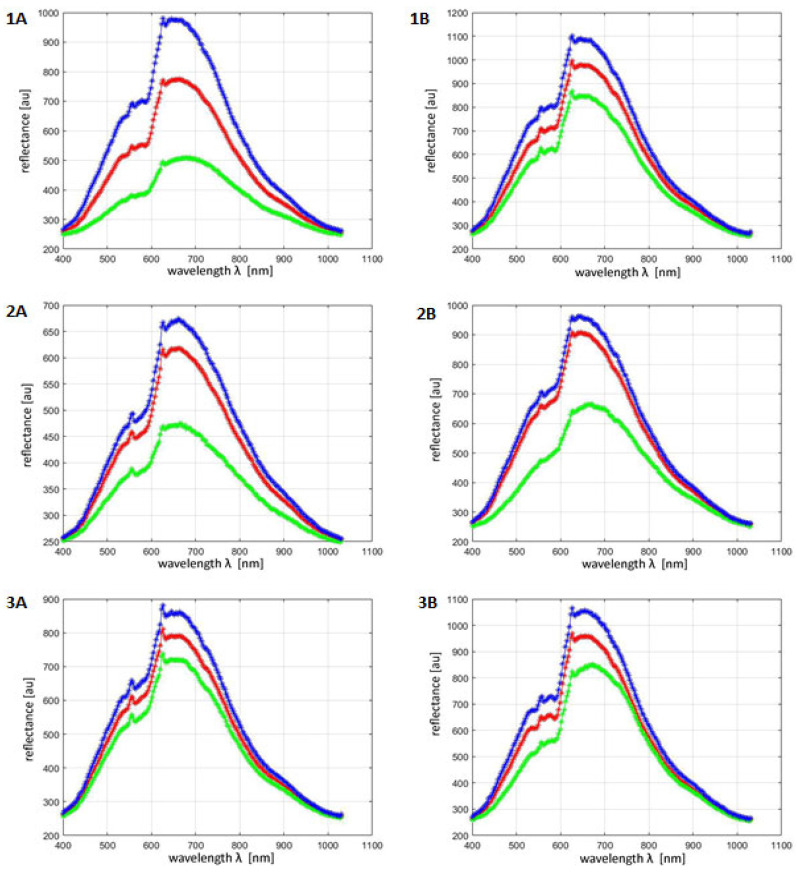
Maximum (blue), average (red) and minimum (green) reflectance before the treatment with kojic acid (patient 1—(**1A**), patient 2—(**2A**), patient 3—(**3A**)) and after the treatment with kojic acid (patient 1—(**1B**), patient 2—(**2B**), patient 3—(**3B**)).

Registration of hyperspectral images allowed for the determination of hyperspectral profiles of ROIs for all tested volunteers before and after the procedure with the use of kojic acid. Figure 3 shows exemplary hyperspectral profiles for volunteers one–three. The blue graph corresponds to the maximum ROI reflectance, the red graph to the average ROI reflectance and the green graph to the minimum ROI reflectance. Since the brightness of a given pixel (after converting hyperspectral images to gray levels in accordance with the algorithm for the external standard) corresponds to its total reflectance, it was possible to automatically identify the brightest pixels (maximum reflectance) and the darkest (lowest reflectance) in the ROI area. Average reflectance was obtained by summing the brightness of all pixels in the ROI and dividing it by the number of pixels.

For the collected hyperspectral profiles, reflectance parameters were determined corresponding to the wavelength of maximum absorption, which was 654 nm. These parameters were then compared with the ROIs of all patients before and after the IPL procedure. The distribution of results was verified using the Shapiro–Wilk test. This pointed to the lack of normality in the distribution of results. Therefore, to compare the values obtained before and after the treatment independently at the wavelength of 654 nm, the Wilcoxon pair order test was used.

At a wavelength of 654 nm, the median mean reflectance value before the procedure using kojic acid was 854 c.u. and after the treatment was 932 c.v.; the increase in reflectance at this wavelength reached the level of statistical significance (*p* < 0.05) (Figure 3).

For each tested volunteer—in order to more accurately assess the effect of the kojic acid treatment on skin reflectance—its range was also calculated, which is the difference between the maximum value and the minimum value in the ROI area at a wavelength of 654 nm before and after the treatment. The ranges of reflectance values determined in this way did not change in a statistically significant way under the influence of the treatment or at the wavelength of 654 nm (*p* = 0.106).

## 4. Discussion

The skin, as the largest organ of the human body, is most exposed to external factors that can directly or indirectly affect its appearance and physiology [26]. It has internal defense mechanisms against external factors. One such defense mechanism is melanogenesis, i.e., the process of enzymatic formation of melanin, which protects the skin against UV radiation [27].

Melanogenesis is regulated by tyrosinase and proteins 1 and -2 (TRP-1 and TRP-2). Tyrosinase, through the hydroxylation of tyrosine to dihydroxyphenylalanine (DOPA), followed by further oxidation of DOPA to DOPA quinone, plays a large role in the production of melanin. Therefore, tyrosinase inhibition helps in achieving hypopigmentation of the skin [28].

Melanin is a complex polymer derived from tyrosine, which is synthesized in the lower layer of the epidermis by melanocytes. Melanocytes are specialized dendritic cells found among the keratinocytes of the epidermis. They play a major role in the production of melanin in melanosomes and spread to nearby keratinocytes. In the process of oxidation and other complex reactions, melanin can change into black–brown eumelanin, or yellow–red pheomelanin, which cause color changes in the human population [29,30].

In many cases, overexposure of the skin to melanogenesis stimulators can cause hyperpigmentation of the skin. Depending on the depth at which the skin pigment accumulates, epidermal hyperpigmentation, dermal hyperpigmentation and mixed dermal–epidermal type are distinguished [3,4]. Hyperpigmentation is an umbrella term for various skin discoloration, pigmentation and darkening disorders [1]. In epidermal hyperpigmentation, there is increased pigmentation of the epidermis, while in dermal hyperpigmentation there is marked pigmentation within the dermis and reduced pigmentation of the epidermis [2]. Cutaneous epidermal hyperpigmentation is characterized by an increased melanin content in the epidermis and dermis. Examples of this type of hyperpigmentation are post-inflammatory hyperpigmentation resulting from injuries caused, for example, by strong peels or laser therapy, and hyperpigmentation caused by inflammatory dermatoses, including acne. Epidermal hyperpigmentation is the most susceptible to topical treatment due to the relatively shallow location of the lesions [3,4]. Pigmentation is regulated by genetic, hormonal and environmental factors that modulate the distribution of melanins in the skin. Melanin protects against UV radiation. Discoloration can be caused by an increase in melanin deposited in the epidermis or dermis.

Heavy metals can also cause discoloration after the long-term use of drugs containing bismuth, arsenic, silver or gold [31].

Metal-containing drugs, especially when applied topically, have a high potential to activate tyrosinase. Heavy metals cause increased pigmentation, partly due to the deposition of metal particles and partly due to the increased production of melanin in the epidermis [32].

Tyrosinase contains a copper ion, which, in contact with UV rays, enhances its activity. KA prevents the activation of tyrosinase by capturing the copper ion, which can prevent the formation of melanin [33].

The pigmentation process is regulated both by external factors, such as UV (ultraviolet) radiation, drugs and substances obtained from plants, as well as internal factors such as endocrine, paracrine and autocrine factors [34].

In addition, excessive skin pigmentation may be affected by an increase in the amount of skin chromophores, which are the group of atoms that give color to the light-absorbing substance. The main chromophores of the skin are melanin and hemoglobin [11,35].

There are many preparations and techniques used in the fight against skin discoloration. Cosmetologists and dermatologists dealing with skin discolorations can use in therapy, for example: vitamin C, retinoids, acid peels and invasive techniques, e.g., lasers [36]. One of the acids used to fight skin hyperpigmentation is kojic acid, which has been recognized by the FDA—Food and Drug Administration—as an alternative to hydroquinone, which is not allowed in cosmetics in Europe [12].

Kojic acid is a natural metabolite of fungi that can inhibit the formation of melanin due to the inhibition of tyrosinase activity [12].

Kojic acid has also shown inhibition of NF-κB cellular activity in human keratinocytes. NF-κB activation probably contributes to the anti-melanogenic effect that is induced by kojic acid [37,38].

Kojic acid is becoming an object of research in many scientific fields due to its antiviral and antimicrobial effects [39]. Researchers have tested the effectiveness of kojic acid many times, but in most studies they considered combining kojic acid with other acids with a skin lightening effect. In addition, the vast majority of studies are qualitative or semi-quantitative. Among the works in which the authors try to assess vascular changes in quantitative terms, the dominant ones use tools with implemented image analysis and processing algorithms [40,41]; 3D antenna, [42,43]; and Primos [44,45].

Nevertheless, a number of limitations that these tools have should be noted. Firstly, they have already implemented image analysis and processing algorithms that are not disclosed by the manufacturers. Thus, it is not possible to verify whether the proposed methods of image analysis and processing are reliable, repeatable and have satisfactory sensitivity and specificity. Secondly, these methods are not dedicated to specific clinical indications. This makes it necessary to use universal methods of image analysis and processing, which will not be optimal in a wide range of pigmentation changes. Thirdly, these tools are designed for mass sale, which means that they must be relatively cheap and easy to use. As a result, it is difficult to compare a strictly scientific device, such as a hyperspectral camera, with relatively simple tools for image acquisition and analysis [46,47].

In this study, quantitative studies were performed on the effectiveness of 3% kojic acid in reducing skin hyperpigmentation. The assessment of the severity of pigmented lesions was performed using hyperspectral imaging. A hyperspectral camera operating in the spectral range of 400–1000 nm was used, because this range coincides with the maximum absorption/reflectance of skin chromophores, including melanin. It should be emphasized that the performed hyperspectral analyses were quantitative—when registering hyperspectral cubes for each volunteer, an external calibration panel was used to quantify the reflectance in the entire analysis area for the full spectral range. For the collected hyperspectral profiles, the reflectance parameters corresponding to the wavelength of the maximum absorption for melanin were determined—in the studies in question, this was 654 nm. The analysis of the results shows a significantly reduced reflectance coefficient for the wavelength of 654 nm. This may be associated with a decrease in the concentration of melanin in the skin. Reducing the melanin content causes the absorption of radiation to decrease and, thus, the reflectance to increase. Therefore, the proposed method can be proposed as a quantitative method for measuring the melanin content in the skin. The superiority of the hyperspectral method over other spectroscopic methods should also be emphasized. In the classical spectroscopic method, a very small area of the skin is sampled—less than 5 mm^2^. This means that the obtained result may not be representative for a larger area of skin, and there are also difficulties with measuring exactly the same places over a wider time period (e.g., before and after a series of treatments). In addition, the radiation beam in the spectroscope head is directed perpendicular to the skin surface, and therefore the skin response is limited to a very narrow examination angle. In hyperspectral imaging, the above limitation does not occur because image acquisition is carried out in a wide range of reflection angles—to put it simply, in the optical range of the focal length of the hyperspectral camera lens.

Studies obtained using hyperspectral imaging have been confirmed by studies conducted using classic clinical photography in cross-polarized light. Conducting studies using image analysis and processing methods, it was shown that kojic acid reduces skin discoloration: increasing skin brightness by 75%, reducing skin contrast by about 83% and increasing skin homogeneity by about 67%. These results are analogous to the results of research by Deo and colleagues, who in 2013 conducted research on the effectiveness of 1% kojic acid in reducing skin hyperpigmentation and in combination with other substances with a similar effect. Studies have shown an improvement in skin tone in over 58% of people using kojic acid alone. Kojic acid in combination with other substances showed slightly better results in reducing skin discoloration [48].

Regardless of the selection of a preparation to reduce discoloration, an important aspect is the method of assessing its effect. Photographic documentation or subjective expert assessment is often used in research. In this study, a hyperspectral camera was used, which allowed for an objective, accurate and non-contact measurement method. The use of this research technique may contribute to a more accurate assessment of the effectiveness of the treatments performed. Nkengne and colleagues used the SpectraCam^®^ hyperspectral camera as the newest and most reliable method for measuring skin color in their study. They showed that the data obtained through hyperspectral imaging are reproducible, precise and, thanks to the very high resolution of the image, they can be used to check cosmetic procedures, which was confirmed in this study [49]. This type of imaging fills the gap in the imaging of visible light with conventional, known devices using classic CCD matrices. A big problem in skin testing is its segmentation and proper calibration of the obtained results [50].

Due to the lack of publications on the use of hyperspectral imaging for the quantitative assessment of the effect of a preparation containing kojic acid on hyperpigmentation of the skin, it is difficult to relate the obtained results to other literature data. Nevertheless, the use of a hyperspectral camera can be used for further scientific research. The results of this study confirm that hyperspectral imaging can provide a more reproducible and more accurate examination of skin lesions after the use of cosmetic and apparatus preparations.

## 5. Conclusions

Examination with a hyperspectral camera allows for an objective assessment of the results of procedures with the use of medium-depth peels. Kojic acid has been shown to be effective in reducing post-inflammatory post-acne discoloration.

## Figures and Tables

**Table 1:** Mean and standard deviation for skin brightness, contrast and homogeneity before and after kojic acid treatment.

Skin	Brightness	Contrast	Homogeneity
Before applying kojic acid	143.33 ± 23.75	5.86 ±1.09	0.56 ± 0.16
After applying kojic acid	157.67 ± 19.89	5.21 ± 1.06	0.64 ± 0.13

## Data Availability

Not applicable.
